# Pulmonary Embolism (PE) in Transit in Heparin-Induced Thrombocytopenia (HIT) With Negative Serotonin-Release Assay (SRA)

**DOI:** 10.7759/cureus.25868

**Published:** 2022-06-12

**Authors:** Tayyab Cheema, Tri Kieu, Mark Balek, Muhammad Ahmad, Pooja Singh

**Affiliations:** 1 Internal Medicine, Advocate Illinois Masonic Medical Center, Chicago, USA; 2 Internal Medicine, West Suburban Medical Center, Oak Park, USA; 3 Cardiology, West Suburban Medical Center, Oak Park, USA

**Keywords:** in transit, pe in transit, anti-pf4 antibody, pf4, pulmonary embolism, pe, serotonin release assay, sra, heparin induced thrombocytopenia (hit), hit

## Abstract

Pulmonary embolism (PE) is a potentially lethal condition, although frequently diagnosed, and is rarely associated with transit on initial presentation. Heparin-induced thrombocytopenia (HIT) can cause both arterial thrombus formation and venous thromboembolism. The two forms of HIT must be differentiated in order to guide management. We present a complex and unique case of PE in transit secondary to HIT diagnosed in a patient with a negative serotonin assay.

## Introduction

Heparin-induced thrombocytopenia (HIT) is a potentially lethal, immunologically mediated adverse drug reaction to unfractionated heparin (UFH) or, less commonly, low molecular weight heparin (LMWH). There are two forms of HIT known to literature. Type I HIT, seen in 10% to 30% of patients treated with heparin, was described as benign, mild thrombocytopenia occurring in the first two days after heparin exposure. Platelet count spontaneously normalizes, even with continued heparin therapy, and is not associated with an increased thrombotic risk [1, 2]. Type II HIT refers to the antibody-mediated, potentially fatal disorder, now ubiquitously referred to as HIT, in which heparin therapy needs to be discontinued as soon as the diagnosis is suspected [1-3]. HIT is estimated to occur in 0.1-5.0% of patients receiving therapeutic doses of heparin and is associated with a 50% increase in mortality [1-3]. Serotonin-release assays (SRA) are the gold standard for diagnosing HIT. However, there are a small number of case reports highlighting patients with negative SRA and positive heparin-induced platelet activation test (HIPA) [1-3]. This case report demonstrates an example of positive HIPA with negative SRA for HIT with a rare opportunity to capture a pulmonary embolism (PE) in transit.

## Case presentation

Our case describes a 70-year-old man with a past medical history of hypertension, hyperlipidemia, chronic alcoholism, and type 2 diabetes mellitus complicated by peripheral neuropathy admitted to our medical center’s subacute rehabilitation after undergoing workup for a non-syncopal fall at an outside hospital. Upon admission to the subacute rehabilitation, the patient’s complete blood count was remarkable for microcytic anemia with hemoglobin of 12.6 g/dl and platelet count of 140,000 per microliter of blood. The patient also had liver function test (LFT) abnormalities with aspartate aminotransferase (AST) of 141 international units per liter (IU/L), alanine aminotransferase (ALT) of 84 IU/L, and total bilirubin of 3.4 µm/L. 

The patient was admitted to the medical floor for worsening acute on chronic metabolic encephalopathy thought to be hepatobiliary in nature secondary to alcoholism. Precipitous platelet count drop followed from 146 platelets per microliter of blood (mcL) to 40 mcL in the following 10 days. The patient was previously on subcutaneous prophylactic heparin for five days which was held on admission and a HIT panel was ordered.

Hepatitis panel and thyroid-stimulating hormone (TSH) were unremarkable; however, he began developing worsening hyperbilirubinemia prompting hemolysis work up which was significant for elevation of his lactate dehydrogenase (LDH) at 761 units/L and reticulocyte count of 3%. Liver ultrasonography (US) did not demonstrate any evidence of cholelithiasis or cholecystitis. Given that his model for end-stage liver disease score (MELD) of 26 in conjunction with Maddrey's Discriminant Function scoring of 53 suggested a poor prognosis, a gastroenterologist was consulted who recommended a steroid course, lactulose, and rifaximin.

**Figure 1:**
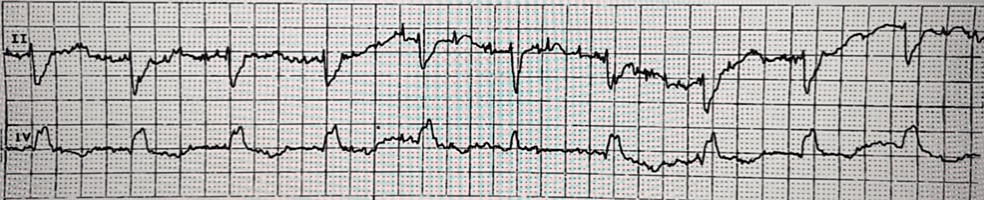
rSr' pattern in setting of a right bundle branch block

**Figure 2:**
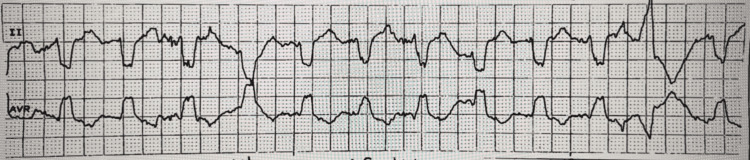
rSr' pattern visualized again

**Figure 3:**
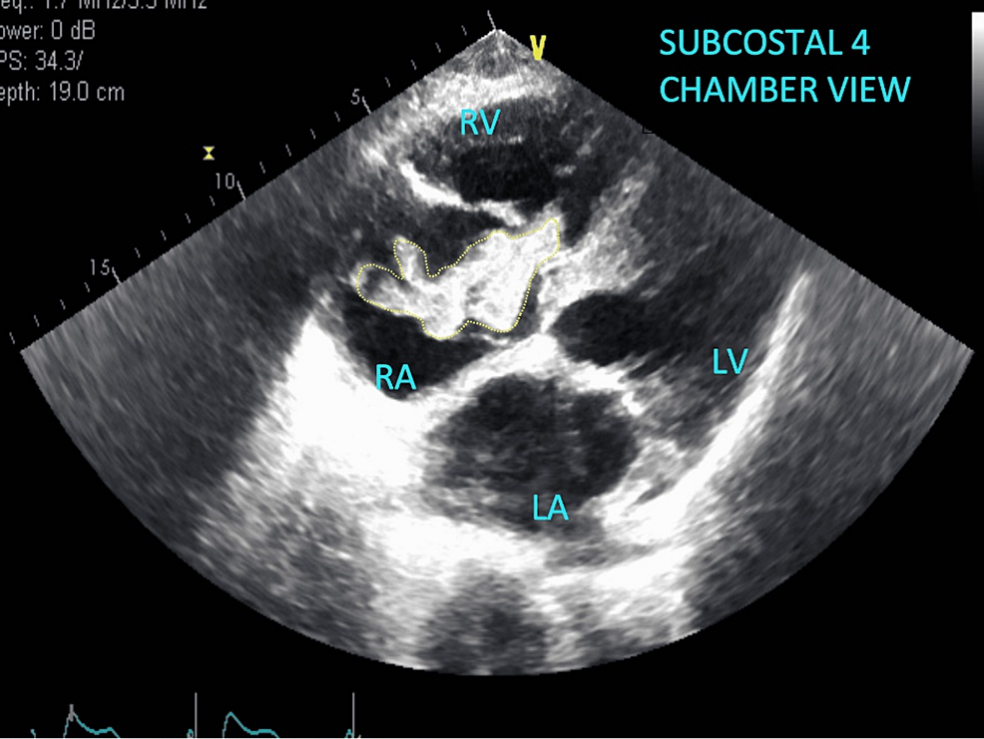
Utilizing the subcostal four-chamber view, a massive thrombus is encased in the right atrium concerning for an impending potential pulmonary embolism RV: right ventricle; LV: left ventricle; RA: right atrium; LA: left atrium

**Figure 4:**
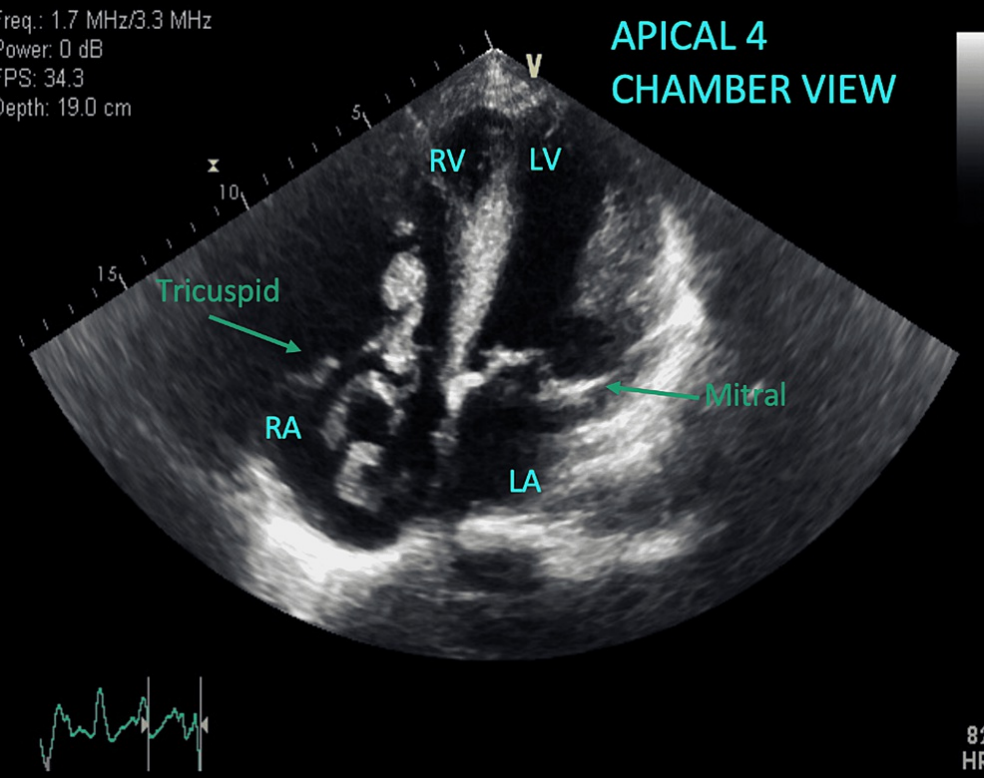
Apical four-chamber view visualizing the large, mobile, serpiginous mass (pulmonary embolus) regurgitating between the tricuspid valve. RV: right ventricle; LV: left ventricle; RA: right atrium; LA: left atrium

The patient’s electrocardiography (ECG) unrevealed a new right bundle branch block (Figures 1-2) prompting evaluation with an echocardiogram which captured a large, serpiginous, mobile mass in the right atrium (Figures 3-4) going back and forth across the tricuspid valve indicative of a PE in transit. 

Furthermore, the positive platelet 4 antibody testing was suggestive of the patient developing HIT. Confirmatory SRA was ordered. Duplex US studies were negative for any lower extremity thrombus, essentially ruling out an embolic phenomenon. Dabigatran was initiated promptly.

Per the input of the interventional cardiology department, the patient was deemed an unsuitable candidate for TPA due to thrombocytopenia as well as for surgery due to multiple medical comorbidities. He was managed conservatively with anticoagulation and discharged to subacute rehabilitation. 

## Discussion

A right heart thrombus in transit is associated with a mortality of greater than 25% and its incidence is reportedly only 4% in all patients with PE [4,5]. Failure to promptly identify a PE in transit has been proven to be highly fatal. PE in transit is classified into three subcategories based on its appearance and echogenic shape. Type A thrombus in transit is described as an elongated, worm-like shape and mobile in nature within the right heart chamber [5]. Type B thrombus in transit is visualized as a firmly attached ovoid mass, while type C is known to have a high degree of mobility and appears to have a combination of characteristics of both type A and B thrombi in transit [5]. Our patient's PE in transit was consistent with a type A thrombus, visualized in Figures 1-2. In a retrospective analysis of 177 cases of right heart thrombus in transit, the mortality rate associated without any form of intervention was 100% [6]. Although current data is suggestive of better outcomes associated with thrombolytic therapy in the setting of hemodynamic compromise or right heart strain (mortality rate of 11% compared to 28.6% with anticoagulation therapy alone vs. 23.8% with surgical embolectomy), there is a paucity of data to guide management with thrombolytics in the setting of HIT [6]. 

This case offers a distinctive presentation of a PE in transit in a patient without an inciting venous thromboembolism phenomenon; instead, involving a rare occurrence of SRA-negative HIT-induced PE in transit associated with an extraordinarily high mortality rate. According to an international pulmonary embolism registry study, only 42 of the 1071 patients (3.9%) with PE presented with thrombus-in-transit through the right heart [7]. Moreover, a higher mortality rate associated with right heart thrombi in PE can be extrapolated; this higher mortality rate compared to PE without thrombus in transit was observed even at three months (reported mortality rate of 29%) [7]. An additional unique aspect of this case revolved around HIT. This patient was found to be positive for heparin-induced antibodies but had a negative SRA. Based on a literature review, mortality amongst patients with HIT-negative SRA appears to be much higher compared to HIT positive SRA [8]. It is possible for patients with a negative SRA but a positive HIT antibody to undergo further testing with PF4-enhanced activation assays such as PF4-SRA. Further data collection and patient case data are required for more standard-of-care use of this test [8]. Here, we present a rare case of SRA-negative HIT-induced PE in transit with complexities reaching beyond the scope of current literature and clinical studies; more data is needed to guide medical management for cases of HIT-induced PE in transit in requiring thrombolytic therapy.

## Conclusions

Management of confirmed HIT can be very difficult and poses an even greater challenge with multiple underlying medical comorbidities, including the possibility of a negative SRA. Currently, there is a paucity of data to guide medical management for HIT-induced PE in transit in patients requiring thrombolytics.
